# European training requirements for the specialty of medical genetics

**DOI:** 10.1038/s41431-025-01899-6

**Published:** 2025-07-02

**Authors:** Ute Moog, Jonathan Berg, Siobhan Kerr, Peter D. Turnpenny, Alain Verloes, Working Group ETR for Medical Genetics, Tina Duelund Hjortshøj, Helen Fryssira, Thomas Meitinger, Béla Melegh, James J. O’Byrne, Feliciano Ramos, Ileana Delia Săbău, Alain Verloes, Fernando Santos-Simarro, Johannes Zschocke, Johannes Zschocke

**Affiliations:** 1 Institute of Human Genetics, Heidelberg University, Heidelberg, Germany; 2 Ninewells Hospital and School of Medicine, University of Dundee, Dundee, Scotland UK; 3 University of Exeter Medical School, Exeter, UK; 4 Department of Genetics, AP-HP-Hôpital Robert Debré, Université Paris Cité Medical School; INSERM UMR 1141 “NeuroDiderot”, Paris, France; 5 Institute of Human Genetics, Medical University Innsbruck, Innsbruck, Austria; 6 Department of Clinical Genetics, University Hospital of Copenhagen, Copenhagen, Denmark; 7 Medical School, National and Kapodistrian University of Athens, Athens, Greece; 8 Institute of Human Genetics, Technical University of Munich School of Medicine and Health, Munich, Germany; 9 Department of Medical Genetics, University of Pécs, Pécs, Hungary; 10 National Centre for Inherited Metabolic Disorders, Mater Misericordiae University Hospital; School of Medicine, University of Dublin (Trinity College); School of Medicine, University College Dublin, Dublin, Ireland; 11 Unit of Clinical Genetics, Service of Paediatrics, Hospital Clínico Universitario ‘Lozano Blesa’, Facultad de Medicina-Universidad de Zaragoza, Zaragoza, Spain; 12 Medical Genetics Department, University of Medicine and Pharmacy Carol Davila, Bucharest, Romania; 13 Departament Genetică, Synevo Laborator de analize medicale, Bucharest, Romania; 14 Unit of Molecular Diagnostics and Clinical Genetics, Hospital Universitari Son Espases, Health Research Institute of the Balearic Islands (IdiSBa), Palma, Spain

**Keywords:** Health care, Social sciences

## Abstract

The development of European training standards is a major contribution to the improvement of medical training at the European level and one of the core activities of the European Union of Medical Specialists (*Union Européenne des Médecins Spécialistes*, UEMS). European Training Requirements for the specialty of Medical Genetics (ETR-MG) were first developed and approved in 2017. Here we report the content and objectives of the 2023 revision, carried out by the ETR-MG Working Group and the officers of the UEMS Section of Medical Genetics in close cooperation with the ESHG and the European Board of Medical Genetics (EBMG). New aspects concern an update of the description of the specialty, the integration of the examination for the European Certificate in Medical Genetics and Genomics (ECMGG), a complete restructuring of the syllabus, and the adaption to novel concepts of medical education.

## Introduction

The European Union of Medical Specialists (UEMS, *Union Européenne des Médecins Spécialistes*) is a non-governmental organisation representing national associations of medical specialists in the European Union and associated countries (https://uems.eu). It is the oldest European medical organisation, created in 1958, and currently consists of 41 member countries. The UEMS works through 43 specialty sections plus a variety of multidisciplinary joint committees and thematic federations. A section of Clinical Genetics was established in 2013. In 2018 the section name was renamed ‘Section of Medical Genetics’ (UEMS-SMG) in accordance with annex V of Directive 2005/36/EC. The aims of the Section, in line with those of UEMS in general, are the harmonisation and raising of professional standards with respect to the training, assessment, practice, and continuous medical education of medical geneticists across Europe. The SMG currently comprises 50 national delegates from 29 member countries; of these, 26 are full UEMS members, whilst Turkey, Ukraine and Georgia are associate members. More information about UEMS-SMG is available at its website https://uems-genetics.org.

UEMS contributes to the improvement of medical training at the European level through the development of European training standards in the different medical disciplines. The development of European Training Requirements (ETR) has been a major activity of many specialty sections and other UEMS bodies; once established, ETRs should be revised every 5 years. UEMS also promotes standardised, high-quality assessment of training by the development of European examinations. The Council for European Specialists Medical Assessment (CESMA) is an advisory body of the UEMS aiming to provide recommendation and advice on the organisation of European examinations for medical specialists at the European level. The Council conducts formal appraisals of European examinations that are valid for 5 years.

Once the SMG had been established, a first version of the ETR complemented with a Description of the Specialty and a Syllabus was prepared by Béla Melegh and Alessandra Renieri, SMG President and Secretary respectively at that time, together with other colleagues. The ETR and Syllabus were closely aligned to the UK’s training programme and syllabus, and was finally approved by the UEMS Council in 2017.

In 2019, an examination to gain a European Certificate in Medical Genetics and Genomics (ECMGG) was introduced as end-of-training exam at the European level. It consists of a written part with multiple choice questions (MCQs), and an oral part comprising short structured oral assessments (SOA). Since 2021, the exam is offered fully online once a year. The ECMGG was developed by the SMG in close collaboration with the ESHG (European Society of Human Genetics) and the EBMG (European Board of Medical Genetics), and has been recently fully appraised by the CESMA. It is regarded as a central component of the endeavour to ensure high quality training in Medical Genetics in Europe. A separate article in this issue provides more details about the ECMGG [1]; further information is also available at www.uems-ecmgg.org.

The recommended revision of the ETR for Medical Genetics (ETR-MG) began in 2021 under the lead of Johannes Zschocke, Chair ETR-MG Working Group, and Ute Moog, President SMG since 2021. It involved a fundamental restructuring, with the training requirements, the Syllabus and the description of the specialty now combined into a single document. The revised ETR-MG also serves as the basis for the ECMGG examination and for educational material provided on a European level. It was approved by the UEMS Council in October 2023. In this article, we describe the procedure of revision and the new aspects of the updated ETR-MG, and discuss how it can contribute to the standardisation of training and the improvement of quality in the care for patients and families with inherited/genetic diseases.

## Materials and methods

### Procedure of ETR-MG revision

In 2021, the SMG in cooperation with the EBMG and the ESHG appointed an ETR-MG Working Group that developed the new ETR in close collaboration with three UEMS-SMG officials (see the authors of this article for detail). The Working Group comprised 10 Medical Geneticists throughout Europe, representing countries with a long tradition in training Medical Geneticists (e.g. UK) and countries with a recent (Greece) or still pending (Spain) recognition of the specialty at the time of ETR revision. It included experienced and young colleagues both from the SMG as well as external experts, and both new members as well as colleagues who had worked on the first version of the ETR-MG.

Revision of the ETR-MG first involved an assessment of the differences in the role of Medical Genetics in European countries, reflected in different training strategies and programmes. A number of individuals (primarily the main authors of this article) then prepared the revised ETR and newly structured Syllabus, which was circulated to the whole Working Group for comments and discussed at several UEMS-SMG Meetings in 2022 and 2023. The final version of the ETR-MG was prepared at a dedicated workshop in Dundee/UK on June 09, 2023, immediately before the ESHG Conference 2023 in Glasgow, and was then sent for review to all professional branches of the EBMG, the ESHG Education Committee and the ESHG Executive Board. Feedback and comments were discussed and incorporated.

In line with the UEMS ETR review process requirements, the draft ETR-MG was shared for review three months before the UEMS Council Meeting, October 2023, with all UEMS bodies, in particular all other specialty sections, all National Medical Associations that are members of the UEMS, and the UEMS ETR Review Committee. After consideration and response to their comments, an updated draft was submitted to UEMS, and shared to all Council attendees one month before the Council Meeting. The final version of the ETR-MG was presented on October 21, 2023, at the UEMS Council Meeting in Malta, and was unanimously approved. It is available for download at the UEMS-SMG and UEMS websites https://uems-genetics.org/Education.html and https://www.uems.eu/european-training-requirements.

### Analysis of national training requirements and survey

The ETR-MG Working Group recognised that the scope of practice of Medical Geneticists differs substantially between different European countries: in some countries it is mostly clinical with little involvement in the provision of genetic tests, whereas in other countries, running a genetic diagnostic laboratory is part of specialist responsibilities. This is reflected in different priorities in specialist training. To obtain basic information on the scope of the specialty in European countries, national training requirements available in English or a language spoken by a member of the Working Group were collated and analysed with support of a student assistant (SK). In addition, a survey was sent to the member countries of UEMS-SMG requesting information on the number of medical geneticists in the country, the proportion of clinical and laboratory work during training, and the respective skills to be acquired (Supplementary document).

## Results

National specialist training regulations were available from 17 European countries (out of 26 countries for which contact information was available, including Bulgaria that is a member of UEMS but not SMG) where Medical Genetics was a recognised specialty in 2022. In most countries, Medical Genetics specialist training takes a minimum of 4 years, with the exceptions of Hungary, Austria and Sweden that require 5–6 years of training. In addition, almost all countries require additional postgraduate experience in other medical specialties, usually 0.5–2 years (4 years in the UK). Specialist training in most countries predominantly involves clinical work, although time supposed to be spent in the diagnostic laboratory may range up to 45% (Germany). All national training requirements include similar areas of knowledge within the four principal domains of basics of human genetics and genomics, genomic variability and its functional consequences, genetic laboratory analyses, and medical genetics. Training and assessment in different countries, however, is highly variable and involves portfolio numbers regarding different contents, formal training courses and different types of assessment in different combinations. Training requirements in the majority of countries include practical skills in genetic laboratory analyses, but only ‘knowledge’ in laboratory diagnostics is required in six countries in which Medical Geneticists do not have to be able to run a diagnostic laboratory (UK, Ireland, Netherlands, France, Belgium and Italy). Biochemical genetics is not included in the training regulations of many countries, but three countries require practical skills in this area as well (Poland, Romania, Bulgaria). Some countries also accept a limited amount of research activities as part of medical genetics training. Methodology for assessing trainees varied widely between countries, with some countries using an exam and portfolio approach (including workplace-based assessments) and other countries citing attendance at specialist courses as the requirement.

A response to the survey was received for 16 UEMS-SMG member countries. The estimated number of Medical Geneticists in different countries ranged from ~1 per million population in Spain (which was the only European country in which the specialty was not yet officially recognised at the time of the survey) to ~20 per million in Hungary, with an average of 6–7. All respondents stated that Medical Geneticists in their countries carried out a higher amount of clinical vs laboratory diagnostic work, with the proportion of laboratory work ranging from 5% (UK, Ireland) to 40–45% (Germany, Austria, Norway). They also reported highly variable numbers of non-physician Clinical Laboratory Geneticists (<1 to 24 per million population, average 7–8) and Genetic Counsellors (rare or absent in approximately half of countries, >5 per million in Scandinavian countries and the United Kingdom). The distribution of tasks in the provision of Medical Genetic services also has an impact on the training requirements for Medical Genetics physicians: the teaching of laboratory skills is by necessity a substantial training component in countries where Medical Genetics specialists have extensive laboratory diagnostic duties, whereas in other countries there is a greater focus on training clinical expertise and skills.

Considering the marked variability in the scope of Medical Genetics practice in European countries, it was decided that a modular structure of the ETR-MG with a focus on general aspects in line with UEMS rules, and a structured, more detailed Syllabus, may best serve its purpose for the time being. Table 1 provides an overview on the content of the revised ETR-MG. No concrete recommendations were made for the optimal length of time of specialist training and the required laboratory diagnostic skills, as this depends on national traditions and expectations and needs to take into consideration the availability of non-medical colleagues in the provision of genetic services.

**Table 1:** Structure of the European Training Requirements for the Specialty of Medical Genetics.

**Specialty of Medical Genetics**
**Aims of Medical Genetics**
**European Training Requirements**
*I*. *Training requirements for trainees*
1. Trainees in Medical Genetics
2. Organisation of training
*II Training requirements for trainers*
1. Process for recognition as trainer
2. Quality management for trainers
*III*. *Training requirements for training institutions*
1. Process for recognition as training centre
2. Quality Management within Training Institutions
**Syllabus**
*1. Human Genetics and Genomics*
1.1. Background and general aspects
1.2. Basics of Human Genetics and Genomics
1.2.1. Genome
1.2.2. Genes
1.2.3. Gene regulation and epigenetics
1.3. Cell biology
1.3.1. Cells and organelles
1.3.2. Cell membrane
1.3.3. Major signalling pathways
1.3.4. Extracellular matrix
1.3.5. Cell cycle and cell division
1.3.6. Mutagenesis, genome maintenance and cell death
1.4. Embryology and developmental genetics
1.4.1. Early embryonic development
1.4.2. Twinning
1.4.3. Organ development
1.4.4. Postnatal development and aging
1.5. Genomic variability and its functional consequences
1.5.1. Genetic and genomic variability
1.5.2. Functional variant effects
1.5.3. From genotype to phenotype
1.5.4. Inheritance modes
1.5.5. Mosaicism
1.5.6. Tumour genetics
1.5.7. Genetics at a population level
1.6. Genetic laboratory analyses
1.6.1. Cytogenetic analyses
1.6.2. Molecular genetic analyses
1.6.3. Tumour genetic analyses
1.6.4. Bioinformatics
1.6.5. Proteomics and biochemical genetics
1.6.6. Experimental biology
*2. Medical Genetics*
2.1. General aspects
2.1.1. Settings
2.1.2. Clinical research in Medical Genetics
2.1.3. Ethical, societal, cultural, and legal aspects
2.1.4. Personalised genetic and genomic medicine
2.1.5. Genetics on the web
2.2. Medical genetic consultations
2.2.1. Genetic diagnosis
2.2.2. Probability calculation and statistics
2.2.3. Genetic counselling
2.3. Genetic testing
2.3.1. Indications for genetic/genomic tests
2.3.2. Postnatal genetic testing
2.3.3. Prenatal genetic testing
2.3.4. Genetic screening
2.3.5. Pharmacogenetics and -genomics
2.4. Management of genetic disorders
2.4.1. General management aspects
2.4.2. Non-specific phenotype-targeting treatments
2.4.3. Gene therapy
2.4.4. Gene editing
2.4.5. RNA targeting
2.5. General Skills
2.5.1. Medical skills
2.5.2. Management skills
*3. Genetic Basis of Human Disorders*
3.1. Congenital morphological anomalies
3.2. Neurodevelopmental disorders (NDD)
3.3. Neurological disorders
3.4. Epilepsies
3.5. Neuromuscular disorders
3.6. Psychiatric disorders
3.7. Ophthalmological disorders
3.8. Craniofacial anomalies and ear, nose, throat disorders
3.9. Dental disorders
3.10. Disorders of the heart
3.11. Respiratory disorders
3.12. Hepatological disorders
3.13. Digestive and gastrointestinal disorders
3.14. Renal and urinary tract disorders
3.15. Disorders of sex development and reproduction
3.16. Skin disorders
3.17. Connective tissue and musculoskeletal disorders
3.18. Skeletal disorders
3.19. Endocrine disorders
3.20. Metabolic/biochemical genetic disorders
3.21. Immunodeficiency, autoinflammatory/autoimmune disorders
3.22. Haematological and coagulation disorders
3.23. Tumours and tumour predisposition syndromes

## Discussion

### New general aspects in the 2023 ETR for medical genetics

The ETR-MG follows the recommended 3-part structure dealing with Trainees, Trainers, and Training Institutions, and is preceded by a preamble that focuses on the UEMS in general and the specialty of Medical Genetics in particular. As Medical Genetics has an interface with most other specialties and has progressed enormously during the last decades, it was felt important to rewrite the paragraphs on the description and aims of the specialty. It is hoped that this contributes to a better understanding of the core and scope of Medical Genetics by other specialists.

It was also necessary to start a process of adjusting the ETR-MG to well-established concepts of medical education, namely the Canadian Medical Education Directives for Specialists (CanMEDS) framework and the concept of Competency-Based Medical Education (CBME). CanMEDS is a physician competency framework created by the Royal College of Physicians and Surgeons of Canada that defines the competencies needed for all domains of medical practice and groups them thematically under seven roles [2]. A competent physician integrates the competencies of all seven roles. The goal of the CanMEDS framework is to enhance the standards of physician training and hereby to improve patient care.

CBME was conceived in the 1970s and was integrated worldwide into medical education and training in many forms [3]. Frank et al. defined it as follows: ‘Competency-based education is an approach to preparing physicians for practice that is fundamentally oriented to graduate outcome abilities and organised around competencies derived from an analysis of societal and patient needs. It de-emphasises time-based training and promises a greater accountability, flexibility, and learner-centeredness.’ [4] A Curriculum developed according to CBME has a focus on specific domains of competence as outcomes of training and shows a relative independence of their time in training [3].

The general aims of Medical Genetics have now been described according to the roles of a medical doctor as defined by the CanMEDS framework, namely as: medical expert, communicator, collaborator, health advocate, scholar, leader, and professional. The content of training and training outcome, in particular with regard to laboratory work, have been described as skills and competencies to be acquired during the training period, rather than a specific number of performances to be completed. A competence-based logbook for documentation of the training has been included.

Several aspects beyond the requirements for trainees have been included in the ETR. Regarding the requirements for trainers, the core competencies for trainers have been re-written, and the important point of ‘training the trainers’ has been added as a part of quality management. Regarding the requirements for training institutions, the focus on multi-disciplinary settings has been strengthened.

### Revision of the 2023 medical genetics syllabus

Medical Genetics is concerned with the identification, interpretation and communication of genetic factors that determine or influence health and disease, as well as medical and personal management based on individual genetic information. In the ETR-MG, knowledge and skills required from specialist medical geneticists is now divided into three major areas:

- *Human Genetics and Genomics:* This includes knowledge of function and regulation of genes and the genome, cell biology and pathophysiology, embryology and pre- and postnatal development, genetic-genomic variability and its functional consequences, as well as laboratory methods for the identification and interpretation of genetic variants.
- *Medical Genetics:* This is defined as the application of human genetics in Medicine, and includes a broad range of general concepts, elements of medical genetic consultations including communication and ethics, relevant aspects to be considered in connection with genetic testing, strategies for treatment and management of genetic conditions, as well as general medical skills required for the specialty.
- *Genetic Basis of Human Disorders:* This is divided into 24 clinically defined areas mostly linked to the areas of expertise defined for the 24 European Reference Networks for rare diseases (ERNs) and encompasses a large number of disorders that are relevant or paradigmatic from a medical genetic point of view.

The structure and content of the Syllabus was fully adjusted with the online textbook APOGeE (A Practical Online Genetics e-Education) which is being created by ERN-ITHACA (coordinator Alain Verloes, France) in cooperation with UEMS-SMG (see https://ern-ithaca.eu/documentation/online-genetics-course-apogee/). The ETR-MG and its syllabus have been mutually interlinked with the ECMGG examination, which is included as a European summative, end-of-training examination, and the multiple-choice questions of the examination are based on the items of the Syllabus. The harmonisation of ETR-MG, APOGeE and ECMGG thus contributes to creating a comprehensive structure and programme for training and examination of specialists in Medical Geneticists in Europe and beyond.

### Role of the ETR-MG in providing high standard patient care

As stated in the ETR-MG, ‘the intention of this document is not to impose a European Curriculum but only to act as a guide for all national associations that wish to implement a European Curriculum at the national level.’ As the collated national training requirements and the survey have shown, there is still a wide variation in Medical Genetics training and professional profiles in Europe. The ETR-MG therefore concludes that ‘… the aim of this document is to help the harmonisation of the different processes of Medical Genetics training in all countries whilst promoting the raising of standards across Europe. It is up to each National Association to adopt or adjust it to their national requirements.’ The implementation at a national level, however, is the long-term goal. As the ETR-MG is not widely known so far, it is of utmost importance to raise its awareness by promoting it through the National Medical Associations, the National Scientific Societies, and the ESHG, by publications and presentations at a national and European level.

The ECMGG examination is not to be considered as formal qualification as such, but due to its high standard and quality its renown has increased over the past few years. As a result, the first countries have recognised the examination as part of, or equivalent to, their national examination, or it has been officially recommended (see ref. 1). The Bureau of the UEMS-SMG is available for questions regarding the drafting of training programmes according to European training requirements or the adoption of the ECMGG examination.

### Levels of competence

According to the concept of CBME, knowledge, skills and attitudes to be achieved are listed in the ETR and Syllabus, but the time or number of performances to achieve an objective has not been fixed. So far, the levels of competence have been described in general terms. The grading follows the definitions given in Table 2. An elaboration of the different levels for all specific competencies that should be achieved during training, including a recommendation on methods to assess progression and outcome, remain to be added.

**Table 2:** Definition of competence levels.

1: The trainee has observed, has knowledge of, describes
2: The trainee performs, manages, demonstrates under direct supervision
3: The trainee performs, manages, demonstrates under distant supervision
4: The trainee performs, manages, demonstrates independently

Although CBME is not new and widely known, its implementation has long been hampered by its complexity, diversity and the lack of a consensus on what constitutes a CBME programme. A study from 2019 defines the core components of a common CBME framework that are suitable to evaluate its implementation: outcome competencies, sequenced progression, tailored learning experiences, competency-focused instruction, and programmatic assessment [5]. With regard to Medical Genetics, all five components would need a specific definition reached through consensus following a broad discussion among stakeholders.

Entrustable Professional Activities (EPAs) form a fifth level of competence, and at the same time go beyond as they connect the competency framework to the workplace. EPAs are defined units of professional work that can be fully entrusted to a trainee once he/she has demonstrated sufficient competence to perform the activity unsupervised [6]. It includes the professional being capable of instructing and supervising trainees him-/herself. EPAs combine multiple competencies for a specific unit of practice, and are ‘observable and measurable in their process and outcome, and suitable for entrustment decisions’ [6]. EPAs are specific to the context of the respective medical specialty. The logic of their development follows one, or a combination of several, perspectives, e.g. procedures, disease or patient categories [7]. In summary, EPAs have a tight relationship but are distinct to the CBME concept [8].

EPAs have found their way into the training and/or professional development programmes of many specialties, and the UEMS highly recommends their introduction into the different ETRs. However, to our knowledge only a few national training programmes for Medical Genetics encompass EPAs. This is also true for the current version of the ETR-MG which neither includes EPAs nor alternatively an elaboration of the CBME framework. Substantial groundwork is still missing for the development of suitable EPAs that adequately reflect the work of Medical Geneticists, which was beyond the resources of the current ETR-MG Working Group.

The implementation of EPAs in the training of Medical Geneticists requires in the first instance the identification of representative core activities that realistically reflect relevant clinical and laboratory tasks. They should align with the knowledge, skills and attitudes described in the curriculum. Hence, the implementation of EPAs for postgraduate education in Medical Genetics requires a consensus with regard to the question ‘What should a Medical Geneticist be entrusted to do?’. This will need to be followed by defining ‘learning outcomes’ and ‘training activities’ for the EPAs and their integration into the Curriculum. On doing so, a selection of learning objectives has to be made, as not all objectives of the curriculum can realistically be captured in EPAs [8]. Finally, methods of assessment of EPAs and of entrustment-decisions have to be implemented. The process of developing EPAs may be challenging and require a long time, but both guidelines and practical recommendations have been published [9–11]. The lack of a process to elaborate the CBME framework or to develop EPAs may be owed to the fact that compared to other specialties, Medical Genetics was only recently established as a specialty, shows a particularly rapid, constant evolution, combines a clinical and a laboratory part, and is often practiced in multidisciplinary settings.

## Outlook

Medical Genetics is not a uniform specialty, and the scope of practice particularly regarding clinical and laboratory work differs markedly between and within European countries. We hope that this paper contributes to discussions among national Medical Genetics bodies to continue a path towards harmonisation of training standards in Europe. Within the UEMS Section of Medical Genetics, a new Working Group to prepare the next revision of the ETR-MG will be established and should include an expert in postgraduate medical education. The refinement of the CBME framework or, probably better, the development and implementation of EPAs needs a broad discussion in the field (‘bottom-up’) to gain consensus on the essentials of the duties of a Medical Geneticist. This discussion needs to include a reflection on the core concept of Medical Genetics as a medical specialty, and will inevitably lead to the identification of overlapping differentiating domains compared to related specialties. Such discussions should have a place in national meetings and sessions of the ESHG Annual Conference.

In the long term, we also recommend modular harmonisation of the training requirements for the different professions in Medical Genetics, including physicians, scientists, counsellors, and probably bioinformaticians, as their functions are overlapping. In addition, individuals who have had personal experience with genetic diseases and have interacted with medical geneticists could provide useful insight. Some EPAs may be carried out by persons of different professional training background, which should be recognised in the description and evaluation of these activities. At the same time, medically trained geneticists may deal with different sets of EPAs at different centres and in different countries, which may require a documented ‘specialisation within the specialty’. Establishing well-functioning competent interprofessional teams is a prerequisite for providing adequate services in the rapidly progressing era of genomic medicine. Studying the varying traditions and experiences in different countries will foster the identification and implementation of the best and most proven elements for providing Medical Genetic services throughout Europe.

## Supplementary information

Supplementary information

## Data Availability

Additional data regarding the analysis of national training requirements and the survey are available from the corresponding author on reasonable request.
