# Supplementary material for: European training requirements for the specialty of medical genetics
Source: Eur J Hum Genet. 2025 Jul 2;33(9):1121–6. doi: 10.1038/s41431-025-01899-6 (PMC12402244; doi:10.1038/s41431-025-01899-6)
Supplement: Supplementary file 1 — Supplementary information [file 41431_2025_1899_MOESM1_ESM.docx]

European Training Requirements (ETR) for the Specialty of Medical Genetics:
Questionnaire for UEMS representatives (2023)

*Johannes Zschocke, Chair of the UEMS-MG ETR working group*

*Medical University Innsbruck, Email johannes.zschocke@i-med.ac.at*

Background information

The UEMS Section of Medical Genetics is currently updating the European Training Requirements for the specialty. As we all know, the skills expected from a medical doctor working as Medical Genetic Specialist differ markedly between countries. In order to make sure that the revised ETR will suit the needs in all countries, we require some basic information from your country. Please use this document as a template and return to the email address above; if there is more than one UEMS representative of your country, please provide the information together on one questionnaire. Please use as much space as necessary (additional pages are welcome)

Information provided by (name/s) ……………………………………………………………………………………

# Questions regarding your country

Which country do you represent? ……………………………………………………………………………………

How many Medical Genetics Specialists work in your country …………………………
(medical doctors, rough estimate is sufficient)

How many Clinical Laboratory Genetic Specialists work in your country …………………………
(Scientists, rough estimate is sufficient)

How many Genetic Counsellors work in your country …………………………
(rough estimate is sufficient)

# Please describe briefly what type of expertise is required from Medical Geneticists in your country (e.g. clinical diagnostics, genetic counselling, laboratory diagnostics, other)

# What is the proportion of clinical work vs. laboratory diagnostic work (vs. research, if at all) in Medical Genetics specialist training in your country?

Clinical diagnostics/counselling ………………………… %

Laboratory diagnostic work ………………………… %

Research ………………………… %

Other (please specify) ………………………… %

# Which skills are expected form Medical Genetics trainees in your country? Please link individual skills to the elements of the revised ETR syllabus

# How much time do trainees spend to develop the respective skills in your country?

# How are the respective skills assessed in your country?

# Any other comments?
